# Rheological Behavior of a New Mucoadhesive Oral Formulation Based on Sodium Chondroitin Sulfate, Xyloglucan and Glycerol

**DOI:** 10.3390/jfb12020028

**Published:** 2021-04-28

**Authors:** Tiziana Maria Grazia Pecora, Barbara Ragazzo, Walter Bertin, Alessia Ragonese, Marco Mascagni, Paola Maffei, Rosario Pignatello

**Affiliations:** 1 Labomar SpA, via Nazario Sauro 35/I, Istrana, 31036 Treviso, Italy; tiziana.pecora@labomar.com (T.M.G.P.); barbara.ragazzo@labomar.com (B.R.); walter.bertin@labomar.com (W.B.); 2 Alfasigma SpA, via Ragazzi del ’99, 40133 Bologna, Italy; alessia.ragonese@alfasigma.com (A.R.); Marco.Mascagni@alfasigma.com (M.M.); paola.maffei@alfasigma.com (P.M.); 3 Department of Drug and Health Sciences, University of Catania, Viale A. Doria, 6, 95125 Catania, Italy

**Keywords:** GERD, mucoadhesion, rheology, viscosity, film-forming capacity, epithelial barrier effect

## Abstract

**Background**: The study aimed at assessing the mucoadhesive properties and the barrier effect of a formulation, labelled as AL2106, containing sodium chondroitin sulfate (ChS), xyloglucan from tamarind seed extract, and glycerol, by evaluating the capacity to adhere to a layer of mucin, the rheological synergism and the barrier effect in comparison to the marketed Esoxx One medical device. AL2106 is a medical device distributed by Alfasigma SpA, Italy with REF FTP57 (Manufacturer: Labomar SpA); it is analogous to Esoxx One medical device: the two products are drinkable solutions that, after swallowing, adhere to the esophageal mucosa, protecting it from the corrosive effect of the gastric acid reflux. AL2106 has been conceived to be better performing in terms of duration of the barrier effect compared to Esoxx One. **Methods**: The mucoadhesive properties, rheological behavior, buffering capacity against acidity, and film-forming ability with the resultant protecting effect on esophagus mucosa (caffeine permeation test) was compared between the two products. **Results**: The mucoadhesivity of the formulations was shown in vitro: both remained adherent to a mucin layer, also when the support was rotated by 90°, and when the film layer was washed with water, intended to simulate the washout due to swallowing. AL2106 showed a good buffering efficacy, being able to absorb at least 50% of its weight of 0.03 M HCl while maintaining the pH above 4. The film-forming effect and barrier properties of AL2106 and Esoxx One were confirmed by an in vitro study on reconstructed human esophageal epithelium. A greater film-forming efficacy of AL2106, lasting for at least 5 h, than Esoxx One was observed. Noteworthy, the barrier function of esophageal tissues was shown to be preserved after the application of both formulations. **Conclusions**: The combination of ChS with the mucoadhesive glycerol−xyloglucan complex and other excipients, which contribute to the barrier effect and to mucoadhesion, contained in AL2106, allowed a longer-lasting protective effect than Esoxx One, proving its effectivity and safety for oral use.

## 1. Introduction

Gastro-esophageal reflux disease (GERD), also known as acid reflux, occurs when stomach acid frequently flows back into the esophagus. This backwash (acid reflux) can irritate or damage the lining of the latter, also causing mucosal breaks in the so-called erosive reflux disease (ERD).

It is a sensorimotor disorder associated with impairment of the normal antireflux mechanisms (e.g., lower esophageal sphincter function, phrenicesophageal ligament), with changes in normal physiology (e.g., impaired esophageal peristalsis, increased intragastric pressure, increased abdominothoracic pressure gradient) or, very rarely, excess gastric acid secretion (Zollinger–Ellison syndrome) [1].

GERD is a multifactorial disease widely prevalent around the world, and especially in developed countries of Northern America, Northern Europe, and Oceania, where prevalence reaches 20–25%. Consequently, GERD has significant societal impacts, affecting adversely work productivity and other quality-of-life parameters of patients [1].

The most common symptoms of GERD are heartburn and regurgitation, but atypical symptoms can be observed, such as epigastric pain or chest pain, which may mirror ischemic cardiac pain, or cough and other respiratory symptoms that may mimic asthma or laryngeal problems [1].

Many studies have shown that in patients with GERD, the defensive properties of the esophagus are impaired, even in patients who do not bear macroscopic mucosal lesions (NERD). The disease shows a marked pathophysiological heterogeneity and the esophagus mucosa damage and the consequent perceived symptoms not always are related to an increased reflux of gastric acid into the esophagus.

Despite the evidence of a microscopical or macroscopic alteration of mucosal integrity in many GERD patients, therapy has mainly focused on acid-suppressive drugs. The pharmacological therapy of GERD includes antiacids, proton pump inhibitors (PPIs), antihistaminic agents (H2 blockers), transient lower esophageal sphincter relaxation (TLESR) reducers, and prokinetic agents and pain modulators as adjuvant drugs [2]. Correction of diet and lifestyle are also highly determinant in controlling and reducing the symptoms and consequences of this disease. The efficacy of acid suppressive therapy has been demonstrated, although about 20–30% of patients display inadequate or absent response to the treatment with PPIs. Moreover, these pharmacological remedies exert their action on the stomach area only, although a damage of the esophageal mucosa has been clearly shown in most patients.

An effective aid can be therefore provided by compounds that protect and reinforce the esophageal mucosa by different mechanisms. In particular, agents that create a physical barrier to block the diffusion of acid, pepsin, and bile acids from the stomach across the esophagus can lower the erosive effect of acid and alkali. These compounds may also participate to mucosa repair and ulcer healing; however, ameliorating the reflux symptoms in patients makes them a valid symptomatic strategy in GERD management. In particular, the combination of mucosal protection with acid suppression may contribute to handle patients which do not respond to PPIs or other therapies. Furthermore, such protective compounds might prolong remission periods and therefore delay relapses, which are quite common in GERD patients after PPI withdrawal [3].

Sodium chondroitin sulphate (ChS) is a glycosaminoglycan (GAG) sulphate, composed of an alternating chain of sugars (N-acetylgalactosamine and glucuronic acid). It is normally found associated with proteins, to form a proteoglycan. A chondroitin chain can have over 100 sugars, each of which can bind sulphate ions in varying positions and quantities. ChS is an important structural component of cartilage, providing almost all of the compressive strength. In the human colon, ChS is present in the flaking epithelial cells and in the meat introduced through the diet. These characteristics suggest that ChS is a good candidate to be used also as a carrier for the colonic delivery of drugs [4].

Orally administered ChS reduces pain in patients with osteoarthritis, especially in the longer periods [5,6]. Although the mechanism of this action is still unclear, the most accepted hypothesis is that ChS inhibits the activities of cartilage degradation enzymes, such as collagenase, elastase, proteoglycanase, etc., and prevents damage to the cartilage from nitric oxide (NO) and free radicals [7].

By electrophoretic and enzymatic techniques, Sekino and Murata [8] showed that ChS is a natural constituent of the structure of the esophagus. A protective effect of ChS on an alcohol-induced gastric ulcer in the rat has also been demonstrated [9]. In a double-blind randomized clinical trial, the combination of ChS and hyaluronic acid demonstrated to be effective and safe in the treatment of GERD symptoms [10].

Tamarind seeds (*Tamarindus* *indica*, L.) is a Leguminosae tree with a long tradition of use in nutrition and oriental medicine, and already in the XVI century, they were used as laxatives in western countries [11]. The seeds of the tamarind have a high content of polysaccharides, among which the most represented is a non-starch branched polysaccharide, called xyloglucan, with an average molecular weight of 52 kD, consisting of a cellulose-like (1→4)-linked β-d-glucan backbone, substituted at O-6 by single-unit α-d-xylopyranose side chains, partially substituted by (1→2)-linked β-d-galactopyranosyl residues; glucose, galactose, and xylose units are present at a 3:1:2 molecular ratio [12]. Tamarind seed extract (TSX) is a neutral, water soluble, and biocompatible polysaccharide widely used in pharmaceutical formulations as a thickener, binder, emulsifier, gelling, and suspending agent [13], due to its ability to thicken or gel in the presence of sugar or alcohol, including glycerol (GLY) [14].

Several researches have recently indicated that TSX can display mucoadhesive properties [15,16,17,18], along with its safety after oral administration in special populations [19,20]. The bioadhesive properties, associated with biocompatibility and biodegradability, make xyloglucan a natural polysaccharide suitable for numerous clinical applications that have been recently studied by the international scientific community: protection of the injured oral mucosa (e.g., in radio-induced stomatitis) [21], inhibition of the adhesion of microorganisms to cell epithelium [22], therapy of acute diarrhea [19], skin protection from UV rays [23], corneal protection [24] and adjuvant in corneal dryness therapy, and even production of controlled drug release systems [25].

Moreover, also the combination of TSX and GLY displayed a synergistic enhancement of their mucoadhesive properties [26].

The aim of the current study was initially to verify the mucoadhesive properties of a series of recently developed formulations containing the same amount of three active ingredients (ChS, TSX, and GLY), but different quantity of other excipients. Among the developed formulations, the more adhesive one (labelled as AL2106) was selected for further technological in vitro studies, including the rheological synergism with mucin and the barrier effect showed by the formulation. AL2106 is a Class III medical device distributed by Alfasigma SpA (Bologna, Italy) with REF FTP57; the manufacturer is Labomar SpA (Istrana, Italy). In a recent study, using an acid/peptic (reflux-like)-damaged esophageal mucosa in a porcine ex vivo model, AL2106 showed to significantly reduce the Evans blue dye (EBD) uptake, taken as a marker of mucosal permeability. The barrier effect was shown to be preserve also after mucosal washing [27].

AL2106 features have been compared to those of Esoxx One, an analogous medical device already on the market in many countries. The combination of ChS and hyaluronic acid (as sodium hyaluronate) present in Esoxx One is basically equivalent to the combination of ChS and xyloglucan (TSX) contained in AL2106. These two products are medical devices in the form of a 10-mL drinkable solution; after swallowing, the products adhere to the esophageal mucosa, protecting it from the corrosive effect of the gastric acid reflux. Using some food-grade technological adjuvants, AL2106 has been conceived to be more performing in terms of duration of the barrier effect compared to Esoxx One. The comparison between the two products, AL2106 and Esoxx One, was therefore carried out in the present work by evaluating the rheological behavior, buffering capacity against acidity, mucoadhesive properties, and film-forming ability with the consequent protecting effect on esophagus mucosa.

## 2. Materials and Methods 

### 2.1. Materials

A partially purified mucin type II from pig stomach was used as biological substrate, and was purchased from Sigma Aldrich Chimica srl (Milan, Italy).

TSX was extracted by the seeds of *Tamarindus indica* L. (EINECS/ELNICS number 254-442-6), Leguminosae, of cultivated plants and harvested six months after flowering. A voucher specimen (42121) is deposited at the Herbarium Mediterraneum, University of Palermo (Palermo, Italy). TSX was purchased from Indena (Milan, Italy) as Xylogel^®^; extraction and manufacturing methods are copyrighted and, therefore, reserved. The content in xyloglucan in this extract is higher than 85%. Vegetal glycerin (GLY) was supplied by Agrar srl (Rome, Italy). The tested batches were manufactured by Labomar SpA (Istrana, Treviso, Italy) under Alfasigma’s commitment. Each formulation differs from the other for the amount of some excipients or presence of mucoadhesive polymers, while the amount of the three active ingredients (i.e., ChS, TSX, and GLY) was kept constant (cf. Tables S1 and S2 in Supplementary Materials).

### 2.2. Adhesion Test

The assayed formulations were evaluated for their ability to adhere to mucosa and to resist to a washing action. To mimic the in vivo conditions, 1 mL of a 12% (*w*/*w*) mucin suspension, prepared as described before, was spread onto a plastic surface and then dried at 70 °C in an oven for 45 min.

The formulation (2.5 mL) was homogenously applied onto the mucin layer; after a few seconds, the plastic support was tilted by 90° for 6 min, to let the excess of not adhered formulation slide away. By determining the weight of formulation that remains attached to the film compared to the weight of formulation loaded onto the film, the % of formulation that adheres to the film can be calculated using the following formula:

% of formulation adhering to the film = (g of formulation which remains attached after tilting × 100)/ (g of formulation deposited onto the mucin film).

The amount of formulation that remains attached after tilting was also evaluated based on the surface area of adhesion, using the formula:

mg of formulation remaining attached/cm^2^ = (mg of formulation adhering to the film)/ (film area in cm^2^).

To simulate the washing effect of saliva, the plastic support covered by the mucin layer and bound formulation was soaked for 30 s in a beaker containing 200 mL water, thereafter left in a vertical position for another 6 min. The entire system was then weighed again and the amount of formulation that remained bound after washing was calculated using the following formula:

mg of formulation attached after washing/cm^2^ = (mg of formulation attached after washing)/ (film area in cm^2^).

From the ratio between the initial amount of formulation attached to the film and the amount of formulation that remained attached after washing, the % weight loss or gain can be calculated using the following formula:

% of formulation lost or gained = (mg of formulation attached after washing × 100)/ (mg of formulation attached before washing).

Each experiment was carried out in ten replicates.

### 2.3. Rheological Evaluation

#### 2.3.1. Sample Preparation

Mucin suspensions were prepared at 6.0% and 12.0% by weight by gently stirring the required amount of mucin powder in water for at least 2 h. Once prepared, all suspensions were used within 4 h, to avoid mucin degradation. For the interactions study, the same amount of 12% mucin and the test formulation were mixed by magnetically stirring for at least 30 min. This led to a final mucin concentration of 6% (*w*/*w*). For the sake of comparison, diluted formulations were prepared by mixing each formulation with the same weight of water under gentle stirring for 30 min. Each experiment was performed in triplicate.

#### 2.3.2. Measurement of Viscosity with the Flow Curve Technique

This technique calculates the viscosity of a sample as a function of shear rate (γ^.^) received from the fluid at a constant temperature. An Anton Paar model MCR 101 rheometer was used (Anton Paar Italia srl, Rivoli, Turin, Italy) connected to a personal computer for setting the analysis parameters and for data processing. Cone-plate was used as the measuring system; measurements were made at 37 °C after a 30 s resting time. To avoid evaporation phenomena during the analysis, the cone rims were sealed with silicon oil. Viscosity data were collected at a shear rate comprised between 20 and 100 1/s. Three replicates were tested for each sample and three measurements were performed for each replicate. Experimental data are gathered as Pa·s as means and variation coefficient (Cv).

#### 2.3.3. Rheological Measurement by the Amplitude Sweep Technique

This method measures the G′ (elastic) module and G″ (liquid) module when the sample is subjected to a variable strain (γ). The test was performed using a cone-plate measurement system (∅ 50 mm) at 37 °C. To avoid evaporation phenomena during the analysis, the cone rims were sealed with silicon oil.

#### 2.3.4. Formulation-Mucin Interaction Studies

Using the same techniques above described, tests were also performed to verify if there were any interactions of the test formulations with a 12% *w*/*w* mucin solution. A formulation/mucin interaction can be registered if the rheological characteristics of the formulation/mucin mixture (at a 1:1 weight ratio in this study) are greater than the sum of the separate rheological characteristics of the formulation and the mucin suspension (i.e., rheological synergy effect).

Therefore, the flow test was used to check whether there was any interaction between the test formulations and mucin by comparing the viscosity profiles of a 1:1 formulation/mucin mixture with those of the individual components at the same concentration they would have in the mixture.

The possible rheological interaction was verified through to the following formula [28]:

$$\Delta \eta \%=[\eta_{mix}-\eta_{f}+\eta_{m}/\left(\eta_{f}+\eta_{m})\right]\times 100$$

where Δ*η*% is the rheological synergism on viscosity, *η*_*mix*_ is the apparent viscosity of the 1:1 formulation/mucin mixture, *η*_*f*_ is the apparent viscosity of the tested formulation diluted with water (1:1 by weight), and *η*_*m*_ is the apparent viscosity of a 6% mucin dispersion (i.e., having the same concentration as in the mixture).

In the Amplitude Sweep technique, a rheological interaction between the tested formulation and mucin can be demonstrated if G′ and G″ values of their mixture are greater than the sum of single G′ and G″ values of each component (diluted formulation and diluted mucin). Such an effect indicates a rheological synergism and can be calculated as follows:

ΔG′ = G′_mix_ − (G′_f_ + G′_m_), and

ΔG″ = G″_mix_ − (G″_f_ + G″_m_)

where ΔG′ and ΔG″ are the values of rheological synergism of the two modules, G′_mix_ and G″_mix_ are the modules of the 1:1 formulation/mucin mixture, G′_f_ and G″_f_ are the modules of the diluted formulation, G′_m_ and G″_m_ are the modules of a mucin suspension at the same concentration of mucin contained in the mixture with the formulation.

Noteworthy, the above calculation is possible only if a linear viscoelastic range is observed for all the experimental measurements.

### 2.4. Evaluation of Buffering Potency of AL2106

After measurement of the pH value of the formulation sample (20 mL), 0.4-mL aliquots of 0.03 M HCl (pH 1.44) were gradually added under magnetic stirring and the pH of the resulting mixture was measured. A curve of the amount of acid added versus the relative pH was plotted in order to evaluate the buffering effect of the formulation.

### 2.5. Evaluation of the Film-Forming Capacity and Barrier Effect

A Reconstituted Human Esophageal Epithelium (HO2E/S/5; batch 17-HO2E-001) (Episkin, Lyon Cedex, France), with an area of 0.5 cm^2^ and an approximate thickness of 100 µm was employed. The model reproduces the Esophageal Epithelium morphology: an epithelium is formed after 5 days of airlifted culture of K510 human epithelial cell line (derived from squamous cell carcinoma) in a chemically defined medium on inert polycarbonate filters. The received material was preliminarily tested for the absence of HIV, *Hepatitis B*, *Hepatitis C*, and *Mycoplasma*. The maintenance medium (SMM, Episkin, Lyon Cedex, France) was tested for sterility. The inserts containing the tissues at day 5 were shipped at room temperature in a multiwell plate filled with an agarose nutrient solution in which they were embedded. Tissues were then removed from the agarose solution in a sterile air flow cabin. The inserts were rapidly transferred to a 6-well plate previously filled with maintenance medium (1 mL/well) at room temperature (21 ± 2 °C) and incubated at 37 °C, 5% CO_2_, under saturated humidity.

As negative control, 30 µL of a saline solution was used (Eurospital, Trieste, Italy); positive control consisted of 30 µL of liquid paraffin (Nova Argentia spa, Gorgonzola, Milan, Italy).

The barrier function and fence properties of the tested formulations were preliminarily assessed by Trans Epithelial Electrical Resistance (TEER) measurement (T = 0 h).

After the initial TEER measurement, 30 µL of the undiluted test products, reference product (paraffin oil) and negative control (saline solution) were directly and uniformly applied on the epithelium at room temperature. To study the percutaneous absorption, the OECD guideline no. 428 recommends using caffeine (MW = 194.2 Da, logP = −0.08) as a reference compound at low lipophilicity. Six replicates for each specimen were made.

After 3 h of treatment, 100 µL of 0.5% caffeine solution in water were applied in the apical (donor) compartment for an exposure of 3 h, without washing out the product. After 1, 2, and 3 h from the application of caffeine solution, the receptor fluid (saline, 1 mL) was collected in plastic tubes and stored at 2–8 °C until the analytical determination. The concentration of caffeine was analyzed by a HPLC/UV-DAD method (see below). Results are expressed as mg of caffeine and shown as % of active penetrated compared to the applied amount and to the negative control.

#### Analytical Method

Caffeine was determined by HPLC (Agilent series 1200 with Chemstation) (Agilent Technologies Italia Spa, Cernusco Sul Naviglio, Milan, Italy) on a reverse phase column (C18) (ZORBAX Eclipse XDB-C18, 5 μm, 4.6 mm × 150 mm). The detection system consisted of an UV-DAD detector set at 275 nm. An eluent mixture of 80% water (added with 10 mM ammonium acetate) and 20% 1:1 acetonitrile/methanol, at a constant flow rate of 1 mL/min, was used, according to the scheme reported in Table S1 (see Supplementary Materials). Under these conditions, the retention time of caffeine was approximately 4.4 min. The above method resulted to be specific and linear in the range 0.1–100 µg/mL of caffeine (r^2^ = 0.9999).

At the end of the caffeine permeation assay and after the receptor fluid collection, the excess of the test sample was removed by washing off and the following endpoints were performed: (i) barrier function and fence properties by TEER (t = 6 h), and (ii) barrier permeability by Lucifer Yellow (LY) paracellular passage (t = 6 h). The whole above protocol was carried out in triplicate.

To evaluate the LY flux, 0.5 mL of die (500 μM in saline) were applied in the apical compartment (into the insert). One mL of saline was added in the basolateral compartment. After 1 h incubation at 37 °C, the transport of LY from the apical to the basolateral compartment was measured. Reading was performed with an Infinite M200 spectrofluorimeter (Tecan Life Sciences, Männedorf, Switzerland) at an excitation wavelength of 428 nm and an emission wavelength of 535 nm. For each tissue, the measurement of fluorescence (RFU) was done at the basolateral level and flux was calculated with the following equation:

LY Flux % = (RFU BL/RFU AP t = 0) × 100

where BL = basolateral; AP t = 0 = apical (mean of the RFU of 500 µM LY). Each analysis was carried out on 3 samples for each batch in triplicate (n = 9).

## 3. Results

### 3.1. Evaluation of Adherence to Mucosa

The ability of the tested formulations to adhere to a mucosal layer and to resist to washing was assessed. With the aim at mimicking the in vivo conditions, the tested formulations were adhered to a mucin-covered surface (which mimics the surface of the esophageal mucosa) and the amount of formulation that remained attached to the surface was evaluated. After adhering to mucin, the formulation was “washed” to mimic the washing action of saliva during physiological swallowing.

The percentage of each formulation that remains attached to the surface become thus an indication of its ability to remain stuck to the esophageal mucosa and withstand the rinsing effect of the saliva.

**Figure 1:**
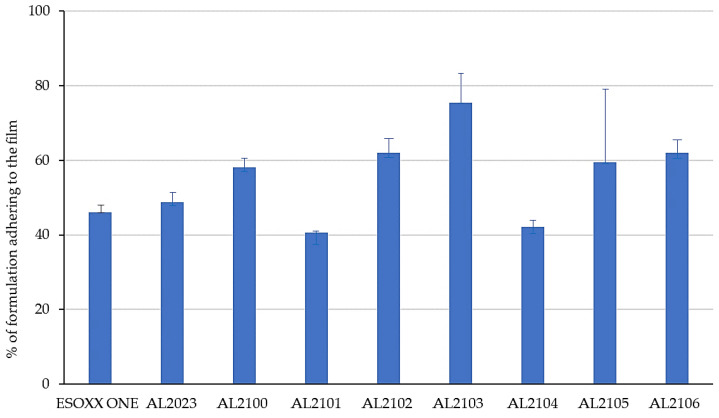
Experimental results of mucoadhesion studies: the bars show the percent of the applied formulation that remains attached to the mucin film after tilting at 90° for 6 min (mean ± S.D. of ten replicates).

**Figure 2:**
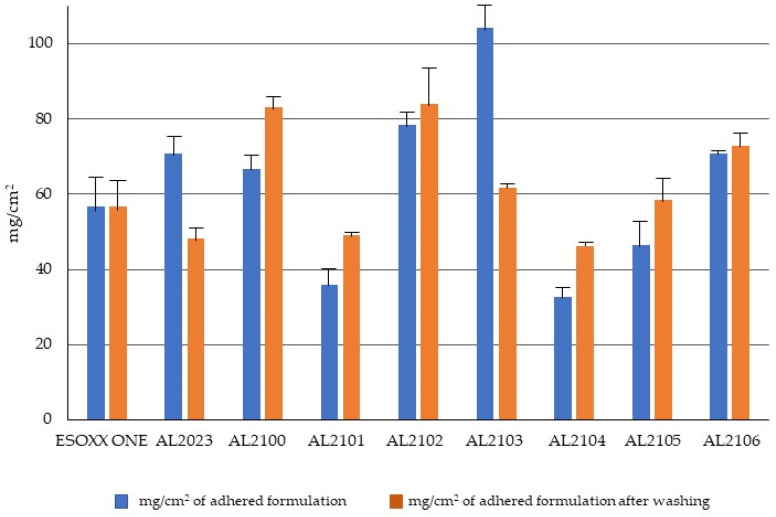
Amount of formulation per surface area unit that remains attached to the mucin film after tilting at 90° and after the subsequent washing in beakers and repositioning at 90° (mean ± S.D. of ten replicates).

Figure 1 shows the % of the formulation that remains attached to the mucin film after tilting at 90° for 6 min. Figure 2 illustrates the amount of the formulation per surface area unit that remains attached to the mucin film after tilting at 90° and after the subsequent washing in beakers and repositioning at 90°.

The formulations that better attached to the mucin film after 90° inclination of the device are AL2100, AL2102, AL2103, AL2105, and AL2106, which showed an adherence of more than 50% of the applied formulation.

After the washing of the formulations in water and the subsequent tilting, some formulations maintained the exact same weight, which indicates that they are able to resist to the washing effect (Esoxx One, AL2102, and AL2106). Some other formulations lose some of their weight after washing (AL2023 and AL2103), indicating that they cannot offer sufficient resistance toward the washing action. Finally, formulations AL2100, AL2101, AL2104, and AL 2105 gained weight after washing; since this phenomenon was due to the formulations’ ability to absorb water, this result must be considered to be negative: by absorbing too much water, these formulations could, in fact, even absorb the gastric reflux.

### 3.2. Rheological Evaluation

A rheological evaluation was performed both on Esoxx One and AL2106 with the aim of highlighting possible different behaviors of the two products in relation to the rheological characteristics. 

**Figure 3:**
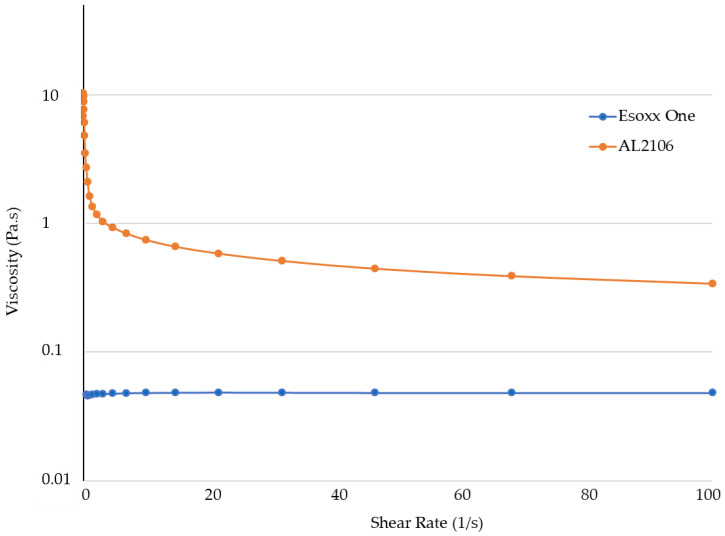
Flow curves (viscosity) of Esoxx One and AL2106 formulations.

**Figure 4:**
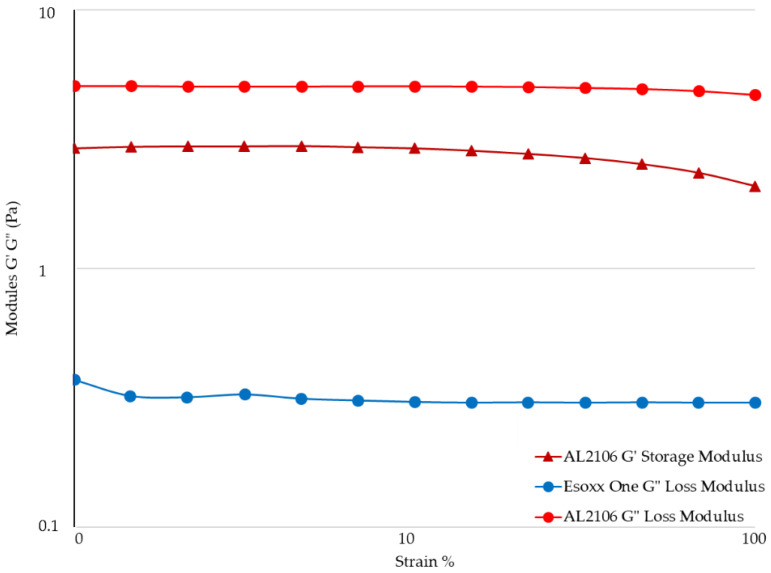
Amplitude Sweep of Esoxx One and AL2106 formulations. G′ and G″ are the elastic and viscous moduli, respectively. The G′ storage modulus for Esoxx One was not measurable.

The viscosities measured in relation to the shear rate at 37 °C are reported in Figure 3, while the value of the G′ (elastic) and G″ (viscous) moduli for the formulations in relation to the strain are shown in Figure 4.

In the flow curves of the two products, which describe the viscosity of the formulation as a function of the applied shear rate, a very different behavior can be noted. Esoxx one demonstrates a Newtonian-like behavior in which viscosity is not affected by the mechanical stress. This type of behavior is typical of products that approach the behavior of water in which the viscosity is steady also when the mechanical stress increases or decreases.

Vice versa, AL2106 shows a viscoelastic behavior: the viscosity of the formulation is not a constant and depends on the mechanical stress. In particular, when the product is stressed, the viscosity decreases; while at rest, under low mechanical stress, the viscosity increases exponentially. The decline in viscosity as shear increases is attributable to the progressive breakdown of the intermolecular network which is present in the product. 

Upon oral administration, AL2106 undergoes a considerable mechanical strain, due to swallowing: during this phase, the product possesses a low viscosity, which is a plus considering the patient compliance. When the product reaches the esophagus, the mechanical strain is very low and therefore the viscosity increases. The increase in viscosity of the product when it reaches the mucosa can be also considered an advantage, because it makes the product more compact and able to adhere to the mucosa.

The rheological measurements by Amplitude Sweep technique described in Figure 4 confirm a different internal structure for the two tested products. It was not possible to determinate the value of G′ of Esoxx One, the value being inferior to the detectability of the instrument. G′ is the elastic value, indicating the presence of an intermolecular network into the fluid: the fact that this module is very low in Esoxx One confirms the Newtonian-like behavior described before. 

Conversely, the G′ modulus of AL2106 was clearly detectable. Moreover, G′ of AL2106 is even higher than its G″ viscous modulus: this behavior is typical of vehicles with a structured internal intermolecular network and also confirms the viscoelastic behavior of the formulation. G″, the viscous modulus, can be considerate as indicator of the contribution to the viscosity of the liquid form of a vehicle: as shown in Figure 4, AL2106 shows a higher G″ value with respect to Esoxx one, in-line with their relative viscosities (cf. Figure 3).

#### Evaluation of the Formulation-Mucin Interaction

**Figure 5:**
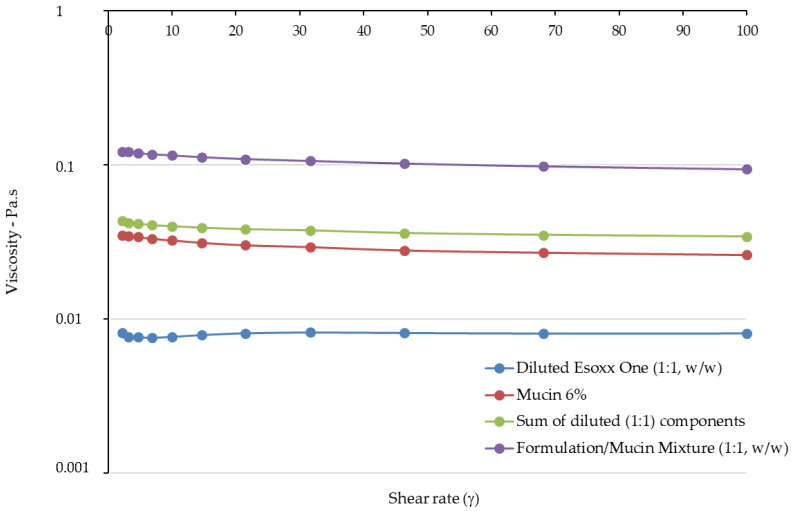
Viscosity of Esoxx One, pure mucin, the sum of the formulation/mucin viscosities, and the viscosity of Esoxx One/mucin 1:1 mixture.

**Figure 6:**
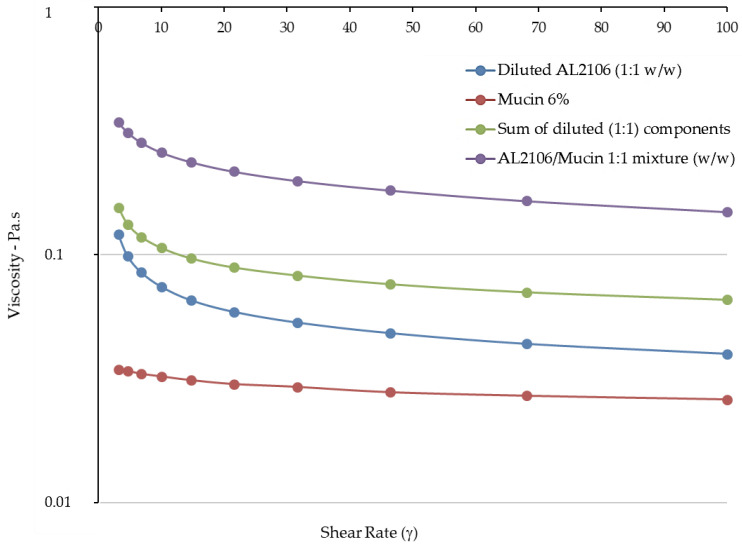
Viscosity of the AL2106 formulation, pure mucin, the sum of the formulation/mucin viscosities, and the viscosity of AL2106/mucin 1:1 (*w*/*w*) mixture.

The viscosity values of Esoxx One and AL2106 formulations, mucin, the sum of their viscosities, and the viscosity measured for each formulation/mucin 1:1 (*w*/*w*) mixture are illustrated in Figure 5 and Figure 6. The rheological synergy value (Δη%) was also calculated to be in the range 174–187% for Esoxx One and in the range 120–145% for AL2106.

In the rheological comparison, the two tested formulations demonstrated to be able to interact with mucin, since for both of them, a rheological synergy occurred, i.e., the viscosity of the formulation/mucin mixture was greater than the sum of the individual viscosity values, respectively, of the formulation and pure mucin. Such a synergy indicates an intimate interaction between the liquid formulation and mucin, suggesting a bioadhesive behavior with the mucin lining the esophageal mucosa.

However, a detailed analysis of the flow curves of the two products, where the viscosity was measured as a function of the shear stress, showed that the two formulations behave very differently. Esoxx One displayed in fact a Newtonian behavior, in which viscosity is not influenced by mechanical stress (shear rate or shear stress); AL2106 had instead a viscoelastic behavior, meaning that its viscosity is dependent on the mechanical stress received; that is, the more stress it receives, the more its viscosity decreases. Moreover, the AL2106 product showed higher viscosity values than those of Esoxx One for every given stress value.

The Amplitude Sweep test can be used to separately determine the characteristics of a semi-solid liquid, by characterizing the elastic-solid modulus G′ (or storage modulus), which is an indication of the solid-elastic response characteristics of a fluid, and the viscous modulus G″ (loss modulus), which is an indication of a formulation’s behavior as a liquid.

**Figure 7:**
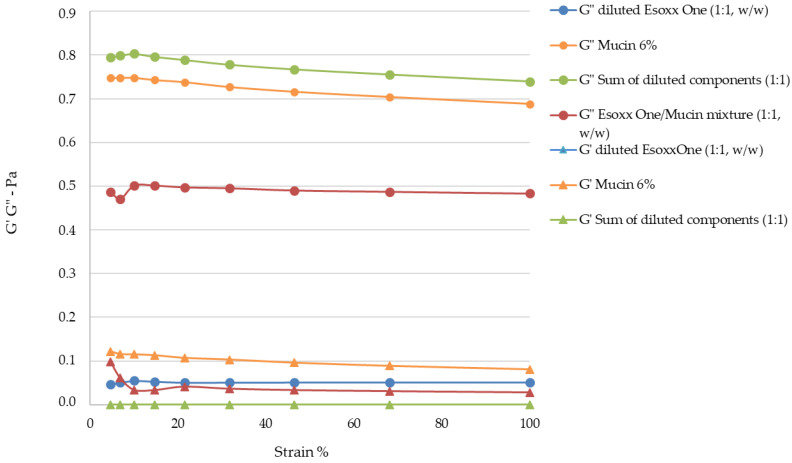
Esoxx One/mucin interaction: G′ and G″ values of diluted Esoxx One, 6% mucin, theoretical G′ and G″ values of the sum of the two formulations and mucin moduli, G′ and G″ values of the Esoxx One/mucin 1:1 (*w*/*w*) mixture.

**Figure 8:**
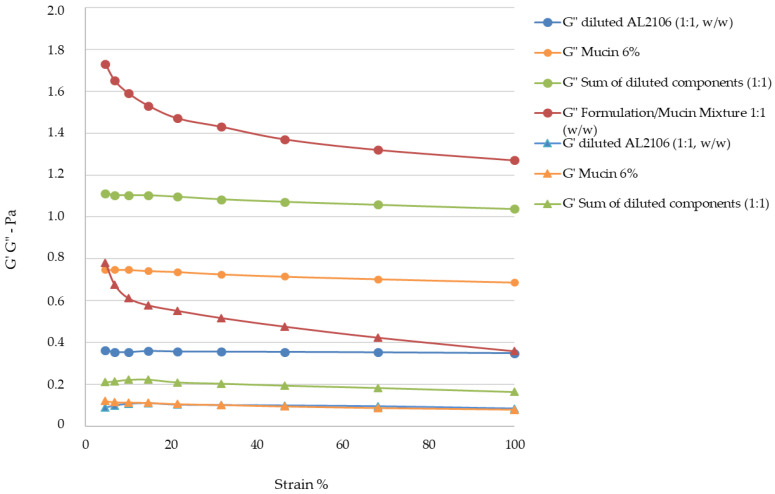
AL2106/mucin interaction: G′ and G″ values of diluted AL2106 formulation, 6% mucin, theoretical G′ and G″ values of the sum of the two formulations and mucin moduli, G′ and G″ values of AL2106/mucin 1:1 (*w*/*w*) mixture.

The G′ and G″ values of the formulations, pure mucin, the theoretical value of the formulation/mucin, and the experimental value of the formulation/mucin 1:1 mixture are reported in Figure 7 and Figure 8.

The analysis of the experimental data obtained using the Amplitude Sweep technique indicated that the G′ value for Esoxx One could not be determined with the available geometry, as its value was below the detection limits. This result reconfirms the formulation’s Newtonian behavior. The G″ values of Esoxx One were also lower than those of AL2106, confirming the fact that the viscosity of Esoxx One is lower than that of AL2106.

Both moduli were able to be determined for the AL2106 formulation, and they indicated that the G′ values never exceed the G″ values, even if the viscosity increased when the mechanical stress decreased (as seen in the flow curve), the elastic modulus never became predominant and the formulation nevertheless continued to behave like a liquid. Furthermore, the fact that the solid G′ modulus can be measured in AL2106 was an indication that this formulation has an own internal structure.

### 3.3. Evaluation of Buffering Potency of AL2106

**Figure 9:**
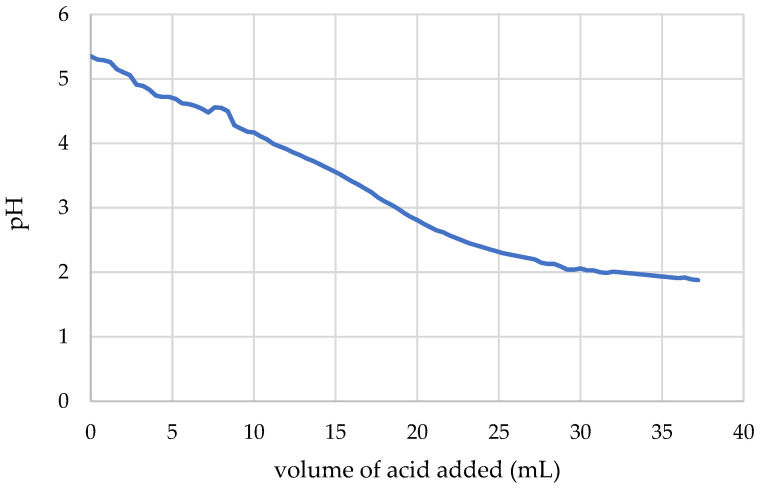
Relationship between the pH of the AL2106 solution and the amount of acid added.

Figure 9 shows the pH values of AL2106 formulation in relation to the volume of 0.03 M HCl added.

The experimental data indicated that the formulation can ensure pH levels that are suitable for protecting the esophageal mucosa. The formulation in fact reaches a pH value around 4 by reacting with about 50% by weight of a HCl solution at pH 1.44, and a pH value around 3 by reacting with 100% by weight of the same acid solution.

Since the gel formulation has a limited ability of absorbing water and loses its adhesive capacity with diluting, the adhering formulation is considered to be able to protect the mucosa from acid reflux.

### 3.4. Evaluation of the Film-Forming Capacity and Barrier Effect

**Figure 10:**
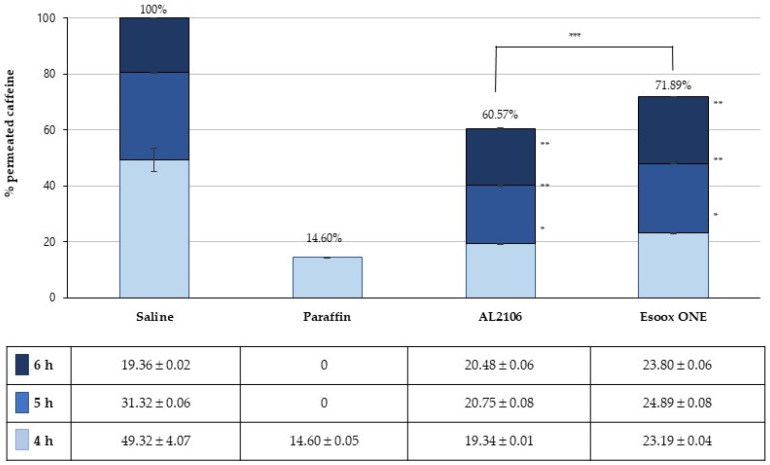
Diffusion of caffeine in the basolateral receptor fluid after 4, 5, and 6 h from the application of the test formulations, expressed as the percentage of the caffeine amount (mean ± S.D. of six replicates) found after the application of saline (negative control). Statistical significance was set at * *p* < 0.005 and ** *p* < 0.0005 vs. saline, and *** *p* < 0.00005 for AL2106 vs. Esoxx One).

The two test products were assayed on a Reconstituted Human Esophageal Epithelium for their film-forming properties based on caffeine penetration. A protocol to assess the film-forming properties on epithelia based on the MEA (Multiple Endpoints analysis) has been developed [29] that includes the modifications induced on barrier integrity (ion paracellular flux by TEER measurement) and tissue permeability (caffeine transit and Lucifer Yellow assay). The products were applied topically on the epithelium surface for 6 h at room temperature and compared to a negative (saline) and a positive control (paraffin) (Figure 10 and Figures S2 and S3).

After 4 h, a significant (*p* < 0.005) inhibition of caffeine passage was associated with the presence of the product on the epithelial surface, with a reduction, with respect to the to the negative control, of 60% for AL2106 and 53% for Esoxx One, respectively (Figure 10). After 5 h, a reduction in caffeine diffusion was still observed (−34% for AL2106 and −21% for Esoxx One). Finally, after 6 h, no difference was found between tissues treated with AL2106 and the negative control, while a 22% increase was observed for Esoxx One (*p* < 0.0005). Values registered at each time point for AL2106 were significantly different vs. Esoxx One (*p* < 0.00005).

**Figure 11:**
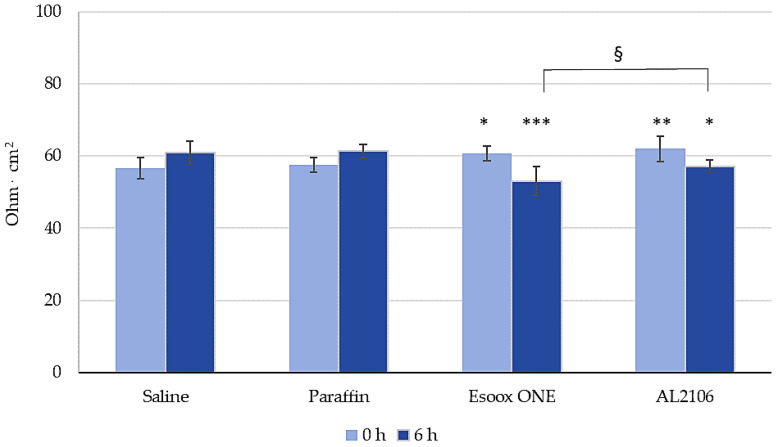
Transepithelial electrical resistance (TEER) expressed in Ohm cm^2^ at baseline (T = 0 h) and after 6 h of treatment (T = 6 h) (mean ± S.D.). Statistical significance was set at * *p* < 0.1, ** *p* < 0.05, or *** *p* < 0.005 vs. saline, and at § *p* < 0.1 for AL2106 vs. Esoxx One.

Figure 11 shows the TEER results expressed in Ohm cm^2^ before application (T = 0 h) and after 6 h of treatment (T = 6 h). TEER was assessed to evaluate the influence of the products on the paracellular flow at the level of the epithelial mucosa. The values before the treatments, ranging from 57 to 62 Ohm cm^2^, correspond to the barrier function associated with both the integrity of the tight joints and the tissue thickness. TEER values recorded after 6 h of treatment with paraffin (positive control) were not different from the negative control (saline) (+7% vs. the TEER measured at T = 0). A slight, although statistically significant, reduction of 8% and 13% was registered after 6 h of treatment with AL2106 and Esoxx One, respectively. After 6 h, AL2106 gave a significant difference (*t*-test, *p* < 0.1) vs. Esoxx One (conversely, the difference measured at 0 h was not statistically significant).

**Figure 12:**
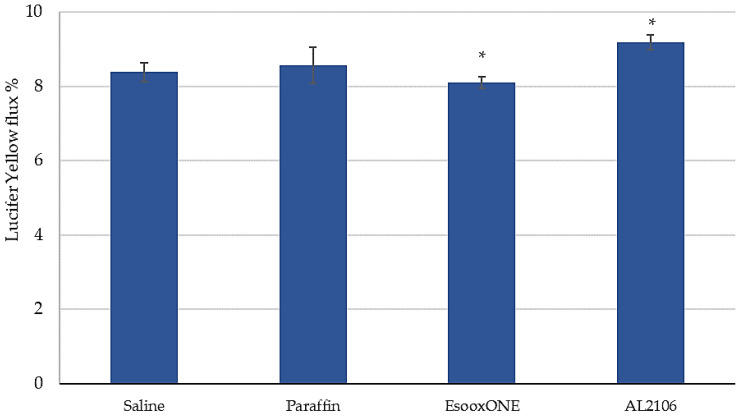
Flow of Lucifer Yellow (mean ± S.D.) after 6 h of treatment followed by washing of the products. Each analysis was carried out on three samples for each batch in triplicate (n = 9) (* *p* < 0.005 for both batches vs. saline and for AL2106 vs. Esoxx One).

The paracellular flow of LY was also assessed at the end of the treatment period and the results of the measured fluorescence are reported in Figure 12, which displays the % LY flow compared to the value at T = 0 in the apical compartment.

The negative control showed a permeability of 8.38% of the applied dose; paraffin application gave a permeability not different from the negative control (8.57%). Only a limited, although significant, difference (*p* < 0.005) was observed between the tested products and the negative control and between the two tested batches.

## 4. Discussion

The aim of the current study was to verify the mucoadhesive properties and the barrier effect of a recently developed oral formulation for the treatment of GERD in comparison with an analogous product already on the market. The formulation, packaged as stick packs, contains chondroitin sulfate, xyloglucan from tamarind seed extract, and glycerol as active ingredients. The interactions between the two components of the formulation and a mucin suspension were investigated as a model mimicking the mucoadhesive properties of the product.

As a first developing step, the capacity to adhere to a layer of mucin was evaluated on a set of AL formulas, differing by some excipients (cf. Table S2, see Supplementary material) and Esoxx One (Table S3) with an in vitro test. All the formulations remained adhered to a mucin layer, also when the support was rotated by 90° and when the film layer adhered to the mucin was subjected to a washing in water, intended to simulate the washout due to swallowing. After dripping the experimental device in a beaker of water and subsequent tilting, some formulations maintained the exact same weight, which indicates that they are able to withstand the washing action (Esoxx One, AL2102, and AL2106); some formulations lost some of their weight after washing, indicating that they cannot withstand the washing action (AL2023 and AL2103). Finally, some other formulations gained weight after washing (AL2100, AL2101, AL2104, and AL2105), indicating that they absorbed water. This property has to be considered as a negative result, since the formulation could be assumed to also absorb the gastric reflux.

In particular, the behavior shown by formulations AL2102 and AL2106 was superior than Esoxx One, because they adhere better to the mucin film and resist to the washing action. Based on these findings and on the recently published ex vivo data on the efficacy of AL2106 to create a barrier protective effect on the esophageal mucosa, similar to that given by the reference product sodium alginate, a protective agent widely used in GERD, and to maintain this effect even after mucosal washing [27], the further experiments were focused on this formulation.

On the selected formulation, rheological studies were performed with the aim to confirm the adhesion capacity by evaluating the interactions between the formulation and the mucin, which represents the major component of the layer that covers the esophagus. The study of the rheological profiles of the formulations, mucin dispersions, and a mixture of tested components and mucin dispersions are representative of the mucoadhesive effect of the ingredients, since it correlates with the so-called rheological synergism [28]. Rheological synergism is defined as an increase in viscosity when a tested formulation and mucus are mixed together: the interaction at the functional group level between the two substrates results in the formation of mixtures that demonstrate greater gel-like properties compared to the same properties of the ingredient and mucin separately examined [30].

Various theories have been propositioned to explain the phenomenon of bio/mucoadhesion, such as the wetting, adsorption, electronic, mechanical, diffusion, and fracture theories [31]. Generally speaking, mucoadhesion can be considered as a two-stage process: initially, the polymer diffuses over the mucus surface (“contact stage”), thereby developing physical interactions (hydrogen bonds, hydrophobic, and Van der Waals forces) and chemical (both ionic and covalent) bonds between the two substrates (“consolidation stage”) [32].

The tested AL2106 formulation displayed a rheological synergy (Δη) with mucin, indicating a mucoadhesion phenomenon [28]. Considering that at physiological pH values, TSX is neutral, while the sialic acids of mucin are negatively charged, a chemical interaction between the substrates is likely to be excluded. Therefore, the polymers and mucin might combine via hydrogen bonds or other non-electrostatic interactions, such as hydrophobic and/or Van der Waals forces, which indeed represent the kinds of interactions appropriate for mucoadhesivity [33]. During the contact stage of mucoadhesion, the polymer chains diffuse in the interfacial region to entangle with the mucin chains, a process that is facilitated by the flexibility of the polymer itself. The addition of GLY may further enhance the mobility and flexibility of the polymer chains, thus favoring their diffusion into the mucus network and eventually assisting the mucoadhesion.

The choice of introducing xyloglucan in the AL2106 formulation was dictated by scientific evidence on its muco-mimetic properties. Mucus is a viscous colloid excreted by the muciparous glands of various body tissues and covers the mucous membranes. It is mainly constituted of water (about 95% by weight) and mucin (up to 5% by weight), an anionic polyelectrolyte. Mucin is a complex glycoprotein made of a peptide backbone and of highly branched oligosaccharide side chains containing residues of galactose, fucose, N-acetylgalactosamine, and sialic acid [34]. Due to its numerous functional groups, mucin molecules can cross-link via various non-covalent mechanisms (hydrogen bonds, electrostatic and hydrophobic interaction, weak Van der Waals forces), allowing its sol-to-gel transition. The ability of mucins to form a gel depends on some variables, like the molecular weight, the fraction of cross-linkable groups, and the ionic strength, and involves solvent-mediated interactions, as the polymer contains both hydrophilic regions (the glycosylated moieties) and hydrophobic amino acids [35], which can produce a physical gelation [36].

In the digestive system, mucus plays many functions: lubricant, to help the passage of food through the intestine; protective of the gastric mucosa from the digestive acids (HCl and pepsin) contained in the gastric juice; defensive, as it entraps bacteria, cellular debris, etc., protecting from external menaces.

The great affinity towards mucin of the xyloglucan−glycerol association, present in the AL2106 formula, can improve the stability of the mucinous film for a prolonged period, without excessively increasing the viscosity of the solution.

Xyloglucan can be defined as a protector of the mucosa, as it stratifies atop the intestinal mucosa to form a bio-protective film, which improves the resistance of mucosa itself towards pathological aggressions and helps restoring its normal functions. In cell cultures, xyloglucan has been shown to increase the Trans Epithelial Electrical Resistance (TEER), an index of good functionality of the tight junctions, confirming its ability to counteract the increase in mucosal permeability induced by exposure to *Escherichia coli* [37].

In this study a comparison was performed between AL2106, as well of some analogous formulations which differ for some side ingredients, and the commercial medical device Esoxx One to evaluate their rheological behavior, measuring the viscosity of the liquid formulation as a function of the shear stress applied. The rheological features of the products were determined by studying its degree of interaction with mucin, as a model of the inner mucosal surface of esophagus. Due to the aims of this work, commercial mucin was chosen instead of freshly prepared mucin specimens due to the lower batch-to-batch variability [38].

The study indicated that the two products are both easily administrable, as they have viscosities between 0.4 and 3 Pa. This value is compatible with most drinkable formulations.

Analysis of the viscosity curves of Esoxx One or AL2106/mucin 1:1 mixture indicated that there was a rheological synergy for both formulations, an implicit indication that an interaction occurred between them and mucin, i.e., of a mucoadhesion phenomenon with the mucosal surface of the esophagus. A linear viscoelastic range (LVE) was not identified in the Amplitude Sweep, which could have been used to compare the moduli, so this technique could not be used to check the interaction between the formulations and mucin. However, evaluating the overall trend of the moduli, we found that only AL2106 exerted a rheological synergy, since the formulation/mucin mixture curves were greater than the sum of the curves of the same formulation and mucin moduli. 

The AL2106 product showed higher viscosity values than those of Esoxx One for every given stress value. Taking in account the purpose of a medical device, the behavior of AL2106 can be considered to be an advantage. In fact, the product must be administered in liquid form and must adhere to the mucin covering the esophagus; the characteristics of the product make it ideal for the ingestion phase where it is subjected to mechanical stresses that reduce the viscosity. The product is also suitable for remaining attached to the surface of the esophagus, since its viscosity is higher when the formulation is not subjected to mechanical stresses.

From the rheological tests performed on the Esoxx One and AL2106 formulations, as well as on their mixtures with mucin, it can be concluded that AL2106 had a greater viscosity than Esoxx One and behave suitably for its administration (in fact, its viscosity decreases when the formulation is subjected to stress, like during swallowing, and is more viscous in the absence of stress, such as when it must adhere to the esophagus wall); furthermore, AL2106 was formulated to have a higher solid elastic modulus than Esoxx One: this indicates a greater intrinsic structuring of the formulation.

The efficacy of an antacid formulation is related to its ability to neutralize the acid stomach fluid and its transit time along the stomach (buffering capacity). The buffering potency of AL2106 was thus determined, showing that it absorbed at least 50% of its weight of 0.03 M HCl while maintaining the pH to values above 4, a value considered compatible to assess the effectiveness of antiacid remedies [39].

The film-forming effect and barrier properties of AL2106 and Esoxx One were confirmed by a study on reconstructed human esophageal epithelium, by monitoring the permeation of caffeine during 6 h of treatment. The integrity of the epithelial barrier after the treatment was also confirmed by TEER and LY flow measurements.

In vitro reconstructed human epithelia models are closer in terms of morphology (multi-stratified epithelium), biochemical, and physiological properties to in vivo human tissues and represent today the most promising alternative to animals, ex vivo explants, and submerged cell monolayers for in vitro efficacy and safety evaluation of topically applied products [40,41]. The biological relevance and predictivity of these models descend from the presence of an organized tissue with different living cell layers, allowing to assess the products topically at realistic clinical doses and exposure conditions. Such 3D tissue models can be valid tools in internal risk assessment, product development, and formulation selection, and are of paramount importance to assess the safety of a finished product, including medical device biocompatibility. Moreover, 3D tissue models represent the only reliable approach available to define and quantify the nature of the mechanism of action of active compound-based medical devices, providing evidence of their superficial action on body barriers, without pharmacological, immunological, or metabolic activity. Finally, this experimental approach, applied to medical devices, is ever more nearby to Directive 2010/63/EU that, concerning the ethical issues about the use of animals in experimental studies, promotes the replacement of animal testing for scientific purposes once alternatives are scientifically available.

Caffeine has been used in many studies to evaluate the permeability in different models of reconstructed epidermis for in vitro percutaneous absorption studies. For its ability to overcome the epithelial barrier, even in the absence of tissue injuries, caffeine has been used as a probe to assess the propensity of a test product to form a protective film. The reduction of caffeine migration through the biological model can be then used as an index of film-forming/barrier property of a material. Due to its film-forming properties, paraffin is used as positive control, while saline can represent a negative control.

The experimental findings of this study suggest a greater film-forming efficacy for the AL2106 formulation, lasting for at least 5 h, with a maximum reduction of caffeine penetration of 39% vs. the negative control after 6 h, compared to the 28% reduction shown by Esoxx One after the same time.

After the caffeine permeation assay (at T = 6 h), the barrier function and fence properties were determined by measurement of TEER [42] and by evaluation of LY paracellular passage. LY is a fluorescent dye impermeable to the cell membrane. It is used to study the paracellular permeability of a compound. When the junctions are intact, LY shows a very low permeability; if the joints are damaged, LY flow will be much higher. Therefore, this assay is used to verify the integrity of cell junctions in the presence of the compound or formulation to be evaluated.

Considering the results of TEER and LY permeation, it is therefore possible to confirm that with the application of both the tested products, the barrier function of esophageal tissues was preserved.

## 5. Conclusions

Strengthening the esophageal mucosal resistance can be a potential therapeutic target in GERD patients. In recent years, medical devices aimed at improving esophageal mucosal defenses are being introduced on the market. These products form a macromolecular complex working as a physical barrier against the noxious components of refluxate, including both acid and pepsin.

In this study, AL2106 has been chosen among other similar therapeutics due to its better physico-chemical properties and its characteristics have been compared to those of Esoxx One, an analogous medical device already on the market in many countries. AL2106 has been shown to form a stable barrier on the esophagus, lasting for at least 5 h. This greater film-forming efficacy of AL2106 demonstrates the new device is better performing in terms of duration of barrier effect compared to Esoxx One. Noteworthy, AL2106 shows a viscoelastic behavior dependent on the mechanical stress received. The device is therefore liquid when swallowed, but rapidly its viscosity increases, making possible the adhesion to the esophageal mucosa. The unique composition of AL2106, made of a blend of chondroitin sulfate with the mucoadhesive glycerol−xyloglucan complex and other excipients that contribute to the barrier effect, allow a longer-lasting protective effect than Esoxx One.

AL2106 product has also shown an excellent tolerability in in vitro cytotoxicity studies. These data, along with the results of ongoing clinical studies, will be the topic of a forthcoming paper.

## Data Availability

The data presented in this study are available on request from the corresponding author.

## Supplementary Materials

The following are available online at https://www.mdpi.com/article/10.3390/jfb12020028/s1. Table S1. Gradient elution scheme for HPLC caffeine determination. Table S2. Composition of the tested formulations. Table S3. Composition of the Esoxx One formulation. Figure S1. Quantification of caffeine in the basolateral receptor fluid after 4, 5 and 6 h from the application (mean ± S.D. of six replicates). Statistical significance was set at * *p* < 0.005 or ** *p* < 0.0005 vs. saline. Figure S2. Amount of caffeine (mean ± S.D. of six replicates) diffused in the basolateral receptor fluid after 4, 5 and 6 h from the application, expressed as the percentage of the applied dose (0.51 mg). Statistical significance was set at * *p* < 0.005 or ** *p* < 0.0005 vs. saline. Figure S3. Diffusion of caffeine in the basolateral receptor fluid after 4, 5 and 6 h from the application, expressed as a percentage vs. negative control (saline) (mean ± S.D. of six replicates). Statistical significance was set at * *p* < 0.005 or ** *p* < 0.0005 vs. saline.
