# Supplementary material for: Rheological Behavior of a New Mucoadhesive Oral Formulation Based on Sodium Chondroitin Sulfate, Xyloglucan and Glycerol
Source: J Funct Biomater. 2021 Apr 28;12(2):28. doi: 10.3390/jfb12020028 (PMC8167776; doi:10.3390/jfb12020028)
Supplement: Supplementary file 1 [file jfb-12-00028-s001.zip › jfb-1181125-supplementary.pdf]

Journal of Functional Biomaterials

MDPI

SUPPLEMENTARY MATERIAL

Rheological Behavior of a New Mucoadhesive Oral Formulation Based on Sodium Chondroitin Sulfate, Xyloglucan and Glycerol

Tiziana Maria Grazia Pecora [1], Barbara Ragazzo [1], Walter Bertin [1], Alessia Ragonese [2], Marco Mascagni [2], Paola Maffei [2] and Rosario Pignatello [3,*]

[1] Labomar SpA, via Nazario Sauro 35/I, Istrana, 31036 Treviso, Italy; tiziana.pecora@labomar.com (T.M.G.P.); barbara.ragazzo@labomar.com (B.R.); walter.bertin@labomar.com (W.B.)
[2] Alfasigma SpA, via Ragazzi del '99, 40133 Bologna, Italy; alessia.ragonese@alfasigma.com (A.R.); Marco.Mascagni@alfasigma.com (M.M.); paola.maffei@alfasigma.com (P.M.)
[3] Department of Drug and Health Sciences, University of Catania, viale A. Doria, 6, 95125 Catania, Italy
* Correspondence: r.pignatello@unict.it

Table S1. Gradient elution scheme for HPLC caffeine determination.

Time (min)	% 1:1 Acetonitrile/Methanol	% Water (+ 10 mM Ammonium Acetate)
0	20	80
7	20	80
10	80	20
15	80	20
17	20	80
20	20	80

Table 2. Composition of the tested formulations.

	AL2023	AL2104	AL2103	AL2105	AL2106	AL2100	AL2101	AL2102
Chondroitin sulfate sodium	✓	✓	✓	✓	✓	✓	✓	✓
Xilogel	✓	✓	✓	✓	✓	✓	✓	✓
Glycerol	✓	✓	✓	✓	✓	✓	✓	✓
Sodium bicarbonate	✓	✓	✓	✓	✓	✓	✓	✓
Xanthan gum	✓	✓	✓	✓	✓	✓	✓	✓
Xylitol	✓	✓	✓	✓	✓	✓	✓	✓
Sodium benzoate	✓	✓	✓	✓	✓	✓	✓	✓
Natural orange flavouring	✓	✓	✓	✓	✓	✓	✓	✓
Citric acid, anhydrous	✓	✓	✓	✓	✓	✓	✓	✓
Potassium sorbate	✓	✓	✓	✓	✓	✓	✓	✓
EDTA	-	-	-	-	✓	✓	✓	✓
Water	✓	✓	✓	✓	✓	✓	✓	✓
Poloxamer 407	-	✓	-	-	-	-	✓	-
Polyvinylpyrrolidone PVP K30	-	✓	-	✓	-	-	✓	✓
Hydroxypropylmethylcellulose	-	-	✓	✓	✓	✓	-	✓

Table S3. Composition of the Esoxx One formulation.

**Esoxx® One**
Chondroitin sulfate sodium
Sodium hyaluronate
Poloxamer 407
Sodium bicarbonate
Polyvinylpyrrolidone
Xylitol
Sodium benzoate
Flavouring
Potassium sorbate
Water

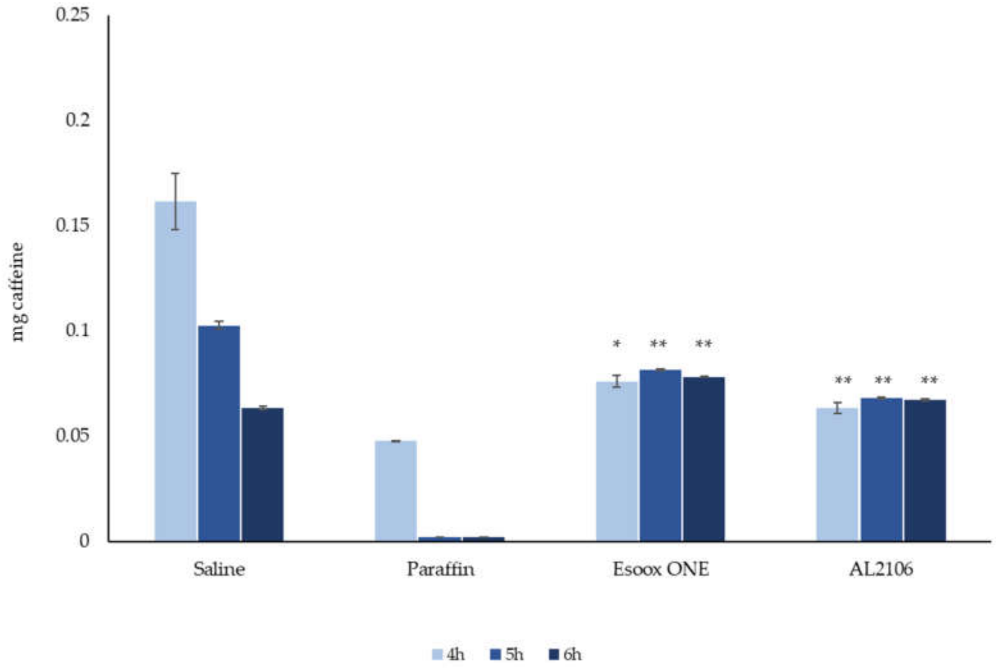

Figure S1. Quantification of caffeine in the basolateral receptor fluid after 4, 5 and 6 h from the application (mean ± S.D. of six replicates). Statistical significance was set at * $p<0.005$ or ** $p<0.0005$ vs. saline.

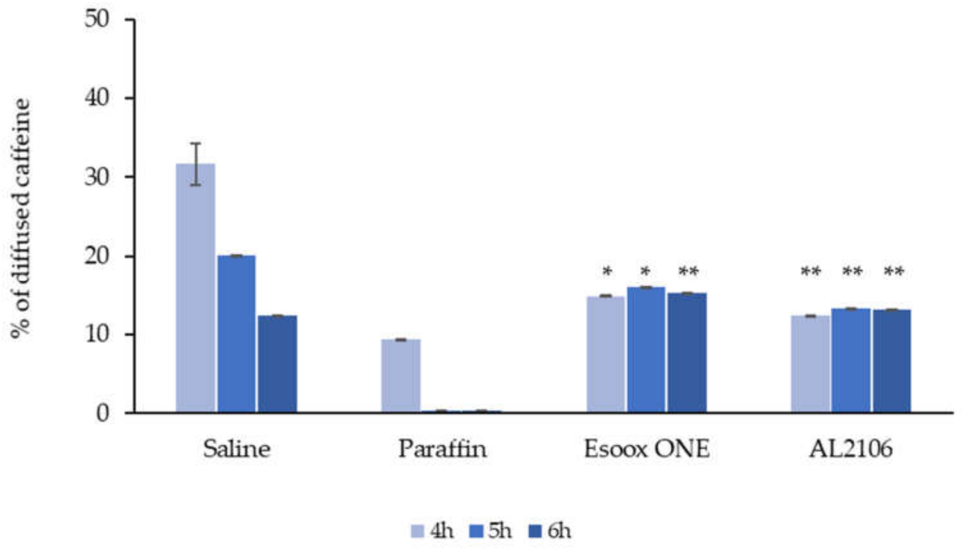

Figure S2. Amount of caffeine (mean ± S.D. of six replicates) diffused in the basolateral receptor fluid after 4, 5 and 6 h from the application, expressed as the percentage of the applied dose (0.51 mg). Statistical significance was set at * $p<0.005$ or ** $p<0.0005$ vs. saline.

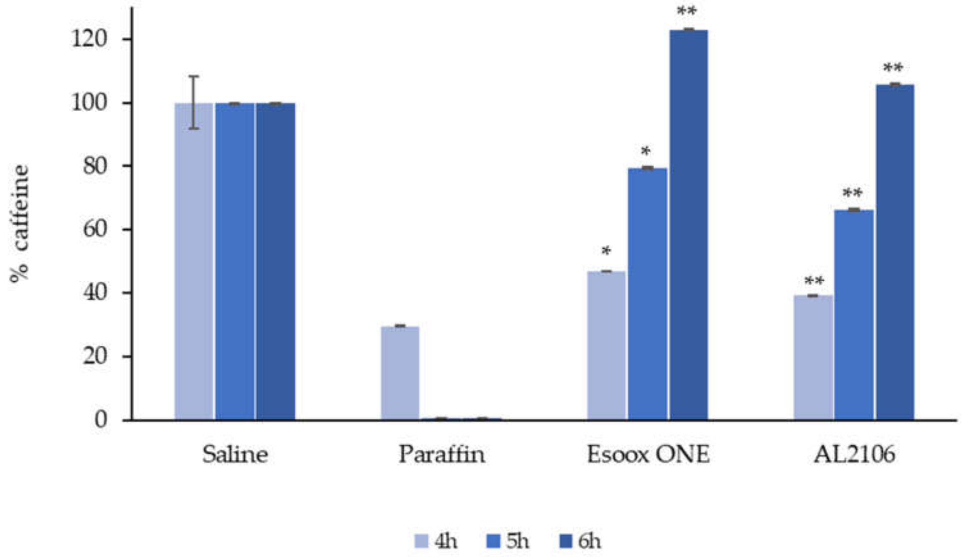

Figure S3. Diffusion of caffeine in the basolateral receptor fluid after 4, 5 and 6 h from the application, expressed as a percentage vs. negative control (saline) (mean ± S.D. of six replicates). Statistical significance was set at * $p<0.005$ or ** $p<0.0005$ vs. saline.
